# Potential of *SP1* as a prognostic marker and therapeutic target in acute myocardial infarction: a bioinformatics, Mendelian randomization and experimental validation study

**DOI:** 10.3389/fimmu.2026.1742618

**Published:** 2026-02-17

**Authors:** Zhongyan Li, Huan Cheng, Jingru Li, Xue Guan, Najie Wen, Luqiao Wang

**Affiliations:** Department of Cardiology, The First Affiliated Hospital of Kunming Medical University, Kunming, China

**Keywords:** acute myocardial infarction, bioinformatics, colocalization analyses, ferroptosis, miRNA-TF-mRNA network, PheWAS, *SP1*, summary-data-based MR

## Abstract

**Background:**

Acute myocardial infarction (AMI) is a global health burden. Ferroptosis drives cardiomyocyte death, but specific ferroptosis-related genes (FRGs) and pathways underlying ischemic injury remain unclear.

**Methods:**

First, differentially expressed mRNAs (DE-mRNAs) from GSE61144 were intersected with FRGs to obtain differentially expressed ferroptosis-related genes (DE-FRGs). Subsequently, GO/KEGG functional enrichment, PPI network construction, expression heatmap visualization and tissue-specific expression were performed on DE-FRGs to clarify their biological characteristics. To explore the potential causal relationship between DE-FRGs and AMI, we conducted summary-data-based Mendelian randomization (SMR) analysis in four cardiovascular-related tissues and performed Bayesian colocalization analysis, ultimately identifying a key transcription factor (TF). With this TF as the hub, a miRNA-TF-mRNA regulatory network was constructed. Based on this pathway, we conducted a series of analyses, including prediction of transcription factor binding sites, GSEA and GeneMANIA analysis, prediction of gene-diseases and gene-drugs associations, phenome-wide association study (PheWAS), ROC curve assessment and RT-qPCR validation, thereby systematically elucidating the molecular mechanisms of this pathway in AMI.

**Results:**

The SMR analysis showed that one key TF-*SP1* were significantly associated with AMI risk among the four types of tissues [coronary artery, atrial appendage, left ventricle and whole blood (*p*SMR < 0.05, *p*HEIDI > 0.05, OR > 1)] in the GTEx database. Bayesian colocalization analysis indicated a strong colocalization relationship between *SP1* and AMI (PPH4 = 0.81). Using *SP1* as the key TF, a miRNA-TF-mRNA network was constructed, including 132 mRNAs and 358 miRNAs. Further narrow down to 2 miRNAs (miR-327 and miR-133b) and 7 mRNAs (*EGR1*, *IL6*, *MYC*, *NR4A1*, *P4HA1*, *PLAUR* and *VEGFA*) through validated by the GSE76604 and GSE4648 datasets. Bayesian colocalization analysis further confirmed that *PLAUR* is the key mRNA and has a strong colocalization relationship with AMI (PPH4 = 0.99). Ultimately, we successfully established the miR-133b/*SP1*/*PLAUR*-ferroptosis signaling pathway. Further analyses provided additional validation for the possibility of *SP1* and miR-133b/*SP1*/*PLAUR* axis as a prognostic marker and therapeutic target in AMI.

**Conclusions:**

In this study, by integrating transcriptomic, SMR and PheWAS analysis, we first established a robust causal association between *SP1* and AMI risk across four cardiovascular tissues, and subsequently delineated a miR-133b/*SP1*/*PLAUR* ferroptosis pathway in AMI.

## 1 Introduction

Acute myocardial infarction (AMI) is defined as an acute coronary event in which a thrombus forms after atherosclerotic plaque erosion or rupture, abruptly interrupting blood flow and precipitating myocardial ischemia and necrosis (1). Despite significant advancements in reperfusion therapy, drug interventions and secondary prevention in recent years, myocardial infarction continues to rank among the top global drivers of mortality and long-term disability (2). Therefore, deep exploration of its pathogenesis, early identification strategies and optimization of treatment plans is of significant clinical and public health importance for reducing mortality and improving prognosis.

Ferroptosis is characterized by the accumulation of iron-dependent lipid peroxides and can be precisely regulated by the *GPX4*-glutathione axis and the System Xc^--^cystine uptake axis (3). *GPX4* serves as a critical antioxidant defense in cardiomyocytes. Under ischemic and hypoxic conditions, its expression is rapidly downregulated, triggering ferroptosis and exacerbating myocardial injury (4). Inhibiting ferroptosis (such as with *GPX4* activators or System Xc^-^ agonists) can reduce the size of myocardial infarction and improve long-term cardiac function (5–7).

Transcription factors (TFs) are proteins that attach to promoter or enhancer regions of DNA, acting as molecular switches to modulate the synthesis of messenger RNA (mRNA) (8). This transcriptional regulation is crucial for ensuring that a given protein is expressed at the proper level in target cells at a particular developmental stage. It not only governs cell proliferation, growth and death, but also regulates the speed of cell migration and tissue formation during embryonic development (9). TFs act as molecular switches regulating the sensitivity of cardiomyocytes to ferroptosis by rapidly reprogramming transcription levels through directly binding to the promoter regions of ferroptosis-related genes. For example, transcription factor *Nrf2* alleviates myocardial damage in myocardial infarction by promoting the expression of *GPX4 (*6, 7).

MicroRNAs (miRNAs) are an important class of non-coding RNA that can specifically recognize and bind to the 3’ untranslated region (UTR) of target mRNA. By pairing with complementary sequences on the target mRNA, they can inhibit its translation or promote its degradation, thereby regulating gene expression and affecting processes such as cell growth, differentiation and ferroptosis (10). Specific miRNAs not only regulate the pathogenesis and progression of myocardial infarction but also hold promise as diagnostic biomarkers and therapeutic targets. For example, miR-432-5p promotes the activation of Nrf2 by binding to Keap1 and regulates the expression of GPX4 and SLC7A11, thereby enhancing the cell’s antioxidant capacity to inhibit ferroptosis (11). In contrast, miR-15a-5p directly targets GPX4, and its overexpression inhibits GPX4 expression, thereby exacerbating ferroptosis and aggravating hypoxia-induced cardiomyocyte injury, regulating ferroptosis in AMI12.

Ferroptosis has emerged as a potential contributor to AMI yet the regulatory landscape of ferroptosis-related genes (FRGs) in AMI remains unclear. This study aims to identify these causal genes by integrating transcriptomic data (GEO) with genetic data from GTEx and FinnGen GWAS, applying Mendelian randomization (SMR), Bayesian colocalization, and network analysis to construct a miRNA-transcription factor-mRNA regulatory axis. We further evaluate the diagnostic and therapeutic potential of prioritized targets through PheWAS, ROC analysis, and experimental validation.

## 2 Materials and methods

### 2.1 Study design

**Figure 1:**
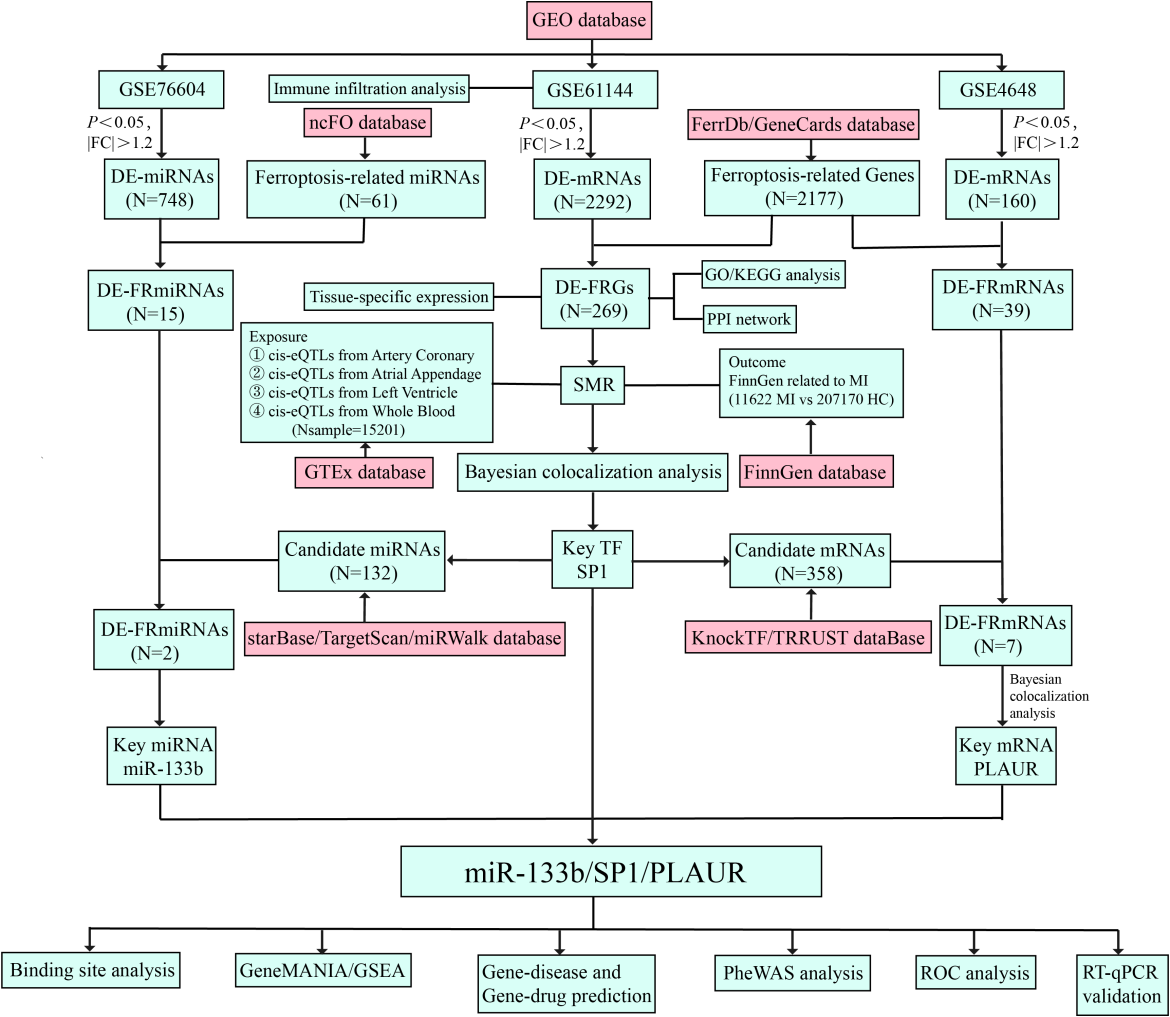
Research design flowchart of this study. MI, myocardial infarction; HC, healthy controls.

This study systematically elucidated the regulatory network of differentially expressed ferroptosis-related genes (DE-FRGs) in AMI. Firstly, based on the Gene Expression Omnibus (GEO) database GSE61144 expression profile, differentially expressed mRNAs (DE-mRNAs) in AMI were screened and intersected with the ferroptosis-related genes to obtain DE-FRGs, followed by GO/KEGG (Gene Ontology/Kyoto Encyclopedia of Genes and Genomes) enrichment analysis, PPI (protein-protein interaction) network construction, expression heatmap visualization, and tissue-expression landscape mapping with FUMA-GENE2FUNC to examine their biological functions. Subsequently, integrating cis-eQTLs data from the genotype-tissue expression (GTEx) project v8 (54 tissues) with the AMI genome-wide association study (GWAS) summary statistics from FinnGen R12 (11622 cases and 207170 controls), we applied summary-data-based Mendelian randomization (SMR) coupled with heterogeneity in dependent instruments (HEIDI) and Bayesian colocalization analysis. These methods identified SP1 (Specificity Protein 1) as the sole DE-FRG whose expression changes show a causal relationship with AMI susceptibility. SP1, a ubiquitously expressed transcription factor that regulates genes involved in inflammation, oxidative stress, and cell survival, has been increasingly implicated in cardiovascular pathophysiology. In this study, we investigated its potential causal role in AMI through multi-omics integration. Upstream miRNAs targeting SP1 were predicted by integrating data from starBase, TargetScan and miRWalk. Downstream targets of SP1 were identified using KnockTF and TRRUST, thereby constructing a comprehensive miRNA-TF-mRNA regulatory network. Further, using the GSE76604 and GSE4648 datasets, differentially expressed miRNAs and mRNAs were screened to pinpoint the miR-133bi/SP1/PLAUR axis. Ultimately, through prediction of transcription factor binding sites, GSEA and GeneMANIA interaction analysis, prediction of gene-diseases and gene-drugs associations, phenome-wide association study(PheWAS), ROC curve and RT-qPCR validation, the regulatory mechanism, diagnostic value and therapeutic potential of this axis in AMI were clarified. **Figure 1** summarized and illustrated the overall workflow of this study.

### 2.2 Ethics declaration

This study utilized publicly available datasets, no human subjects or tissues were directly involved.

### 2.3 Data sources

The mRNA and miRNA expression profiles of AMI (GSE61144, GSE4648 and GSE76604) were obtained from the GEO database (https://www.ncbi.nlm.nih.gov/geo/) (12–14). The number of samples included in each dataset and the corresponding platform information are described in detail in **Table 1**. A total of 2177 ferroptosis-related genes were obtained from the FerrDb (http://www.zhounan.org/ferrdb/current/) (15) and GeneCards (https://www.genecards.org/) (16) databases. 61 ferroptosis-related miRNAs were obtained from the ncFO (http://www.jianglab.cn/ncFO/) (17) database.

**Table 1:** Details of the relevant gene expression profiles applied in this study.

Dataset	Platform	Control	MI	Coutury	Submission	Samples	Application
GSE4648	GPL81	24	36	USA	2006	Heart tissue	Identification for DE-mRNAs
GSE61144	GPL6106	10	14	South Korea	2015	Peripheral blood	Identification for DE-mRNAs
GSE76604	GPL16384	74	23	Japan	2016	Heart tissue	Identification for DE-miRNAs

### 2.4 Identification of DE-mRNAs and DE-miRNAs

Differentially expressed mRNAs (DE-mRNAs) and miRNAs (DE-miRNAs) were identified using GEO2R (https://www.ncbi.nlm.nih.gov/geo/geo2r/), with a significant threshold of *P*<0.05 and |log_2_ Fold change (FC)| > 0.263 between AMI and control samples. GEO2R is a free online tool developed by National Center for Biotechnology Information (NCBI) specifically for performing differential expression analysis on gene expression data stored in the GEO database. Visualization of DE-mRNAs was achieved through volcano plot. Then, by intersecting DE-mRNAs with ferroptosis-related genes (FRGs) and DE-miRNAs with ferroptosis-related miRNAs, and visualizing them with Venn diagrams, we identified differentially expressed ferroptosis-related genes (DE-FRGs) and miRNAs (DE-FRmiRNAs), respectively.

### 2.5 GO and KEGG enrichment analyses of DE-FRGs

To explore the specific mechanism by which DE-FRG drives AMI pathology, we performed Kyoto Encyclopedia of Genes and Genomes (KEGG) pathway and Gene Ontology (GO) enrichment analyses to identify the significant pathways with Metascape (https://metascape.org) (18). Results were visualized as bar and bubble charts.

### 2.6 Construction of protein-protein interaction networks

The PPI network can cluster gene sets into tightly connected modules based on protein interaction strength and identify potential hub proteins through network topology metrics. Therefore, the STRING (https://cn.string-db.org/) database was used to construct the PPI network to find key DE-FRGs.

### 2.7 Heatmap and tissue-specific expression profiling of DE-FRGs

Based on the GSE61144 dataset, the top 50 DE-FRGs with the highest fold changes were selected for heatmap visualization to compare their expression differences between the control and MI groups. Furthermore, their expression distribution characteristics in 54 GTEx tissues were evaluated using FUMA-GENE2FUNC (https://fuma.ctglab.nl/gene2func).

### 2.8 Immune infiltration analysis

Immune infiltration analysis was performed to examine immune microenvironment changes in AMI. Specifically, the CIBERSORT tool was used to analyze the immune cell infiltration abundance of 22 immune cells in the GSE61144 expression profile dataset (19). Additionally, Using the three major R packages “ggpubr, corrplot, and reshape2”, we visualized the results of the immune infiltration analysis: stacked bar charts showed the proportion of different immune cell subsets in each sample, correlation heatmaps systematically analyzed the interaction network among immune cells, and boxplots displayed the differences in various immune cells between the control and MI groups to identify immune cells with significantly different expression in the MI group.

### 2.9 SMR analysis

Mendelian randomization (MR) is a statistical method that uses genetic variants as instrumental variables to infer potential causal relationships between exposures and outcomes (20). Summary-data-based Mendelian randomization (SMR) extends the concept of MR, serving as an efficient genetic analysis method that integrates summary statistics from GWAS and cis-expression quantitative trait loci (cis-eQTL) information, significantly enhancing research efficacy (21). SMR similarly adheres strictly to the three core assumptions of Mendelian randomization: (1) Relevance Assumption: genetic variation is robustly and significantly associated with the exposure factor to avoid “weak instrument variable” bias; (2) Independence assumption: genetic variation is independent of any confounding factors; (3) Exclusivity assumption: genetic variation affects the outcome only through the exposure pathway, and there is no horizontal pleiotropy pathway independent of the exposure (22). Compared with traditional MR, SMR utilizes cis-QTL instrumental variables and summary data, providing higher statistical power in independent large samples. The accompanying HEIDI test distinguishes linkage from pleiotropy, enabling causal inference across multi-omics layers such as gene expression, methylation, and protein abundance. In the SMR analysis, we corrected for multiple testing using the Benjamini–Hochberg (BH) procedure to control the false discovery rate (FDR). SMR p-values for all exposure–outcome associations were adjusted using the p.adjust function in R with the BH method. Associations with an FDR-adjusted p-value < 0.05 were considered statistically significant. SMR is better suited for publicly available GWAS/QTL data and has become an important method for efficiently dissecting gene-phenotype mechanisms (21).

The databases for exposures and outcomes were obtained from different sources, which can reduce the possibility of population overlap and minimize potential bias in MR estimates (23). For exposure, we selected cis-eQTLs data from four types of tissues (coronary artery, atrial appendage, left ventricle and whole blood) in the GTEx database (https://www.gtexportal.org/) (24), which is a large-scale genomics project dedicated to exploring how genetic variation influences gene expression across human tissues. We selected these four tissues based on their key role in the pathogenesis of AMI, as well as the strong evidence from previous studies in the field of cardiovascular genomics. In the GTEx database, coronary arteries are the main sites for the formation and rupture of atherosclerotic plaques (25, 26); heart left ventricle is the main myocardial region affected by ischemic injury, with gene expression reflecting cardiac remodeling and dysfunction (27); heart atrial appendage, while not directly injured in AMI, is closely linked to post-AMI complications such as atrial fibrillation (28); whole blood captures systemic inflammatory processes that drive plaque instability and thrombosis, and is frequently used in cardiovascular disease diagnosis (29). Together, these tissues comprehensively represent the vascular lesion site, affected myocardium, and systemic immune environment, providing a robust pathophysiological foundation for our SMR analysis. For outcoms, the four GWAS datasets related to AMI were acquired from the FinnGen database (https://www.finngen.fi/en/access_results) (30) and IEU Open GWAS (https://opengwas.io/), detailed characteristics of outcome datasets are shown in **Table 2**. In this study, we used the FinnGen AMI dataset as the discovery cohort and validated our findings in three additional independent AMI datasets.

**Table 2:** Description of the AMI outcome datasets employed in this study.

OpenGWAS ID	Trait	Population	Year	Ncase	Ncontrol	Nsnp
finn-b-I9_MI	Myocardial infarction	European	2021	12801	187840	16380433
ukb-e-I21_CSA	Acute myocardial infarction	South Asian	2020	374	8502	9805094
ebi-a-GCST011364	Myocardial infarction	European	2021	14825	2680	10290368
ebi-a-GCST011365	Myocardial infarction	European	2021	14825	44000	8106745

In this study, we used the SMR method to explore the potential causal relationship between DE-FRGs and AMI using SMR software (version 3.1.1 https://yanglab.westlake.edu.cn/software/smr/) (21). We retained significant SMR probes based on an FDR-corrected *P* < 0.05 and required the HEDI *P* > 0.05 to indicate the absence of heterogeneity. Key parameter settings were as follows: Gene region definition: The cis-region was defined as ±1 Mb around the transcription start site (TSS) of each gene, consistent with default and commonly used practice in eQTL-based MR studies (31). eQTL screening threshold: Only independent cis-eQTLs with p < 5×10^-8^ in the exposure (eQTL) dataset were used as instrumental variables. This stringent threshold ensures high-confidence eQTL instruments and minimizes inclusion of weak or spurious associations. Additional default settings were applied, including LD reference from the 1000 Genomes Project (Phase 3, EUR population) and HEIDI test with default window size (1000 kb) and LD r² threshold of 0.9 for clumping. We selected four types of tissues (left ventricle, atrial appendage, coronary artery and whole blood) from the GTEx database to perform four SMR analyses. The positive results of the four SMRs were presented using a forest plot. Subsequently, we took the intersection of the positive results of the four SMRs to identify key gene that have a significant causal relationship with AMI in all four tissues.

### 2.10 Bayesian colocalization analysis

We performed Bayesian colocalization analysis using the “coloc” R package to determine whether GWAS and eQTL signals share a common causal variant (32). This analysis computes posterior probabilities for five hypotheses (PPH): H0: no association with either trait, H1: association with only trait 1, H2: association with only trait 2, H3:association with both traits due to different causal variants and H4: association with both traits due to the same causal variant. Following previous literature, we adopted a posterior probability of colocalization (PPH4) > 0.8 as stringent evidence that the expression-associated and disease-associated signals share the same causal variant (33). We used the key gene obtained from the above SMR for colocalization analysis, and visualized the results using the “locuscomparer” R package.

### 2.11 Construction of miRNA-TF-mRNA network

To further explore the regulatory mechanism of the key transcription factor in AMI, we constructed the miRNA-TF-mRNA network. Firstly, upstream miRNAs targeting the transcription factor were predicted with starBase (https://rnasysu.com/encori/index.php) (34), TargetScan (https://www.targetscan.org/vert_80/) (35) and miRWalk (http://mirwalk.umm.uni-heidelberg.de/) (36), while its downstream target genes were identified using knockTF (http://www.licpathway.net/KnockTF/) (37) and TRRUST (https://www.grnpedia.org/trrust/) (38). Next, we performed differential expression analysis using datasets GSE4648 and GSE76604 to obtain DE-mRNAs and DE-miRNAs, respectively, and then intersected them with the predicted mRNAs and miRNAs to further pinpoint the ferroptosis-mediated miRNA-TF-mRNA regulatory pathways in AMI. Finally, we visualized the miRNA-TF-mRNA network using Cytoscape software.

### 2.12 Binding site prediction

To comprehensively analyze the regulatory interplay among miRNA, TF and mRNA, and to pinpoint their potential binding sites for subsequent experimental validation, this study adopted the following bioinformatics workflow. First, the StarBase (https://rnasysu.com/encori/index.php) database was employed to predict the binding sites between selected miRNA and TF. Next, the JASPAR (https://jaspar.elixir.no/) database was used to predict the promoter regions of target mRNA for putative TF-binding motifs. Finally, the predictions from both databases were integrated to construct a systematic miRNA-TF-mRNA regulatory network.

### 2.13 GeneMANIA/GSEA analysis

Hub genes were inputted to GeneMANIA (https://genemania.org/) to retrieve functionally related genes and build a PPI network, along with data on co-expression, co-localization, shared pathways, and protein-DNA or protein-protein interactions (39). In this study, we explored the top 20 functionally similar genes of hub genes and performed functional enrichment analysis. Use the “ClusterProfiler” package to perform Gene Set Enrichment Analysis (GSEA) on each core gene to obtain the important regulatory pathways involved by the genes. The subset of KEGG pathways in the “C2: curated gene sets” database of MSigDB was used as a reference gene set, and the screening threshold was set at an adj. *P*-value < 0.05. Finally, the analysis results were visualized with ridge plots based on normalized enrichment scores (NES) using the “enrichplot” package (40).

### 2.14 Candidate gene-drug and gene-disease prediction

Gene-drug and gene-disease interactions for the two core genes were independently obtained from the CTD (https://ctdbase.org/) database. Entries were ranked by descending inference score, and the top 20 were retained for each gene. The intersections yielded six common drugs and seventeen common diseases.

### 2.15 Phenome-wide association analysis

Through the AstraZeneca PheWAS Portal (https://azphewas.com/) database, the associations between candidate genes and various phenotypes can be systematically assessed, thereby inferring their phenotypic pleiotropy and potential adverse reaction risks as drug targets (41). This database hosts summary statistics for approximately 15,500 binary and 1,500 quantitative traits.

### 2.16 Diagnostic potential of key biomarkers in AMI

ROC curves were constructed using SPSS software based on the normalized expression matrix data from the GSE61144 and GSE76604 datasets to assess the diagnostic performance of key genes in AMI. The AUC (area under the curve) represents the area under the ROC curve and quantifies the overall performance of the diagnostic model. Higher AUC values (closer to 1) suggest better diagnostic performance of DE-FRGs or DE-FRmiRNA for AMI.

### 2.17 H9C2 cardiomyocytes culture

H9C2 cardiomyocytes were purchased from the American Type Culture Collection (ATCC) and maintained at 37°C under 5% CO_2_ in DMEM medium with low bicarbonate containing 10% serum. To model AMI *in vitro*, cells were subjected to oxygen–glucose deprivation (OGD) by replacing the medium with glucose-free, serum-free DMEM and incubating them in a modular hypoxia chamber flushed with 95% CO_2_/5% N_2_ for 4 h at 37°C.

### 2.18 Expression validation of key biomarkers

To validate the biomarkers, reverse transcription-quantitative polymerase chain reaction (RT-qPCR) was performed on H9C2 cardiomyocytes. Total RNA was extracted using the TransZol Up Plus RNA Kit, and cDNA synthesis and qPCR was carried out using the TransScript^®^ Uni Cells to CT 1-Step Probe Kit. β-actin was used as the endogenous reference gene, and the 2^−ΔΔCt^ method was employed to calculate relative expression levels, with statistical significance defined as *P* < 0.05. PCR primers were designed by Sengon Biotech and primer sequences are provided in **Table 3**.

**Table 3:** Primer and sequence.

Genes	Primer	Sequence (5’ to 3’)
SP1	FORWARD	GAAGCAGCAGCACAGGCAGTAG
	REVERSE	GCCAGCAGAGCCAAAGGAGATG
PLAUR	FORWARD	GCACAGAACGGAGCGTGAAGG
	REVERSE	TTGGAAGCCATTCGGTGGTAAGC
β-actin	FORWARD	TGCTATGTTGCCCTAGACTTCG
	REVERSE	GTTGGCATAGAGGTCTTTACGG
miR-133b	FORWARD	TTTGGTCCCCTTCAACCAGCTA
U6	FORWARD	CTCGCTTCGGCAGCACA

## 3 Results

### 3.1 Identification of DE-mRNAs and DE-FRGs

**Figure 2:**
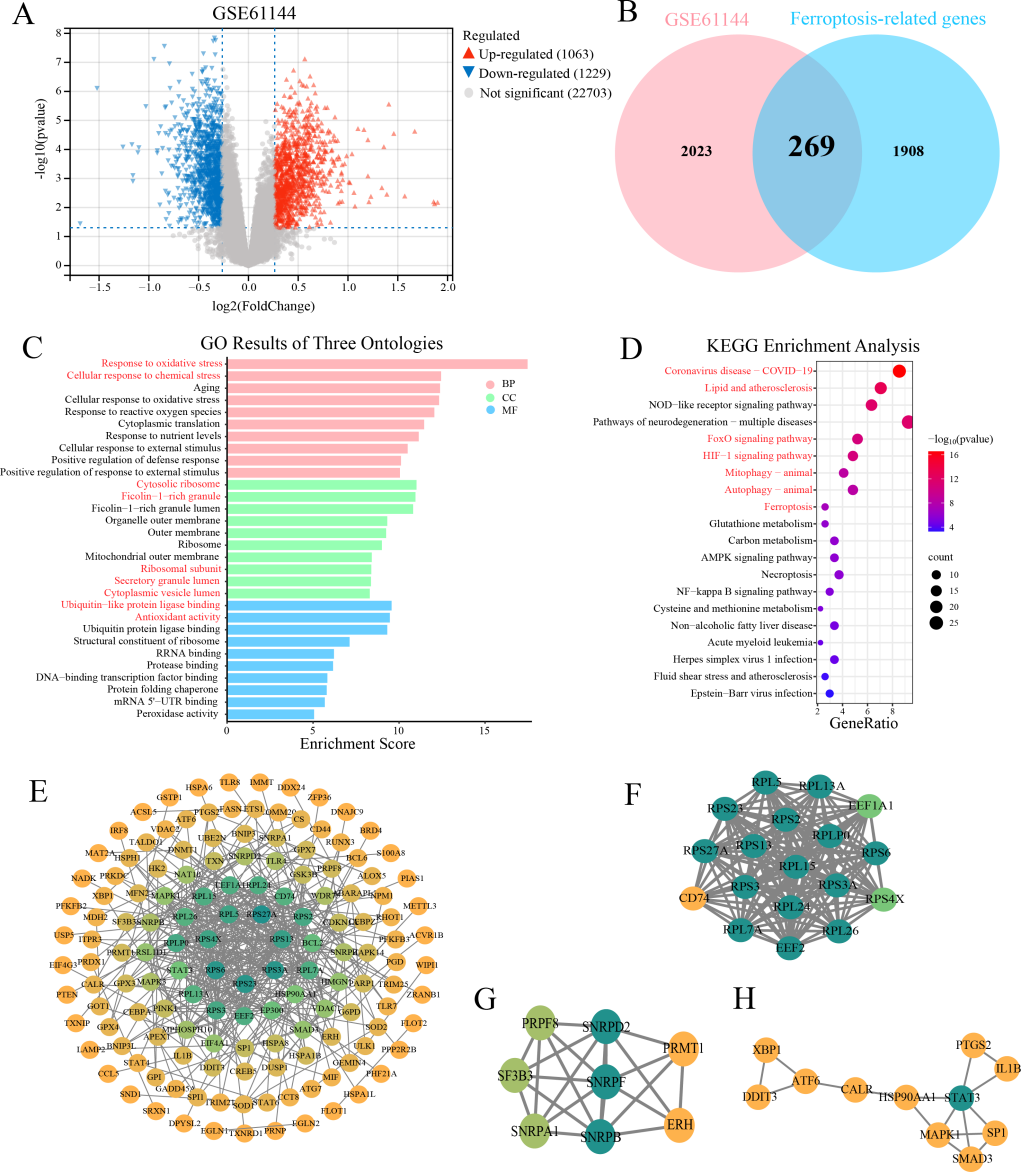
DE-mRNAs analysis, GO/KEGG enrichment and PPI network. **(A)** Volcano plot displays the expression profile of 2292 DE-mRNAs in the GSE61144 dataset. Red triangles represent upregulated genes, gray dots represent non-significant genes, and blue triangles represent downregulated genes. **(B)** Venn diagram illustrates the intersection between 2292 DE-mRNAs and 2177 ferroptosis-related genes. The pink circle represents the DE-mRNAs from the GSE61144 dataset, while the blue circle signifies the ferroptosis-related genes. As depicted in the diagram, there are 269 DE-FRGs. **(C)** Bar chart shows significant enriched GO terms for 269 DE-FRGs. (CC, cellular component; BP, biological process; MF, molecular function). **(D)** Bubble diagram illustrates the significant enriched KEGG pathway analysis of the 269 DE-FRGs. E-H. PPI networks of 269 DE-FRGs and the top three functional clusters. **(E)** Global PPI network constructed in STRING (confidence score ≥ 0.9). **(F)** Cluster 1 (score = 17.765, 18 nodes, 151 edges). **(G)** Cluster 2 (score = 6.286, 8 nodes, 22 edges). **(H)** Cluster 3 (score = 3.2, 11 nodes, 12 edges). Node color represents degree centrality in the PPI network, with a gradient from yellow (low degree) to green (high degree).

In the GSE61144 dataset, 2,292 significantly differentially expressed genes were identified between the AMI and the healthy group, of which 1,063 genes were upregulated in the myocardial infarction group and 1,229 genes were downregulated (**Figure 2A**). By intersecting 2,177 ferroptosis-related genes with 2,292 DE-mRNAs, 269 DE-FRGs were obtained (**Figure 2B**), providing key clues for further exploration of the ferroptosis mechanism.

### 3.2 GO and KEGG pathway analyses of DE-FRGs

GO and KEGG enrichment analyses were performed on 269 DE-FRGs to gain insights into their functional significance. GO enrichment analysis showed that in the biological process category, the DE-FRGs were mainly involved in “response to oxidative stress” (*P* = 3.5E-2) and “cellular response to chemical stress” (*P* = 2.0E-6); in terms of molecular function, “ubiquitin-like protein ligase binding” (*P* = 2.7E-10) and “antioxidant activity” (*P* = 3.4E-10) were significantly enriched; cellular component analysis indicated that the gene products were predominantly located in the “cytosolic ribosome” (*P* = 9.7E-12) and “ficolin-1-rich granule” (*P* = 1.1E-11) (**Figure 2C**). KEGG pathway enrichment analysis demonstrated that the DE-FRGs were significantly enriched in multiple signaling pathways, including lipid metabolism and atherosclerosis (*P* = 9.8E-14), *FoxO* (*P* = 1.7E-11), *HIF-1* (*P* = 2.0E-11), mitophagy (*P* = 2.7E-9) and ferroptosis (*P* = 8.5E-8) signaling pathways (**Figure 2D**). Together, these findings implicate the DE-FRGs in oxidative injury, hypoxia adaptation, mitochondrial quality control and lipid-driven ferroptosis as potential drivers of AMI progression.

### 3.3 PPI network construction

A PPI network was constructed based on 269 DE-FRGs using the STRING database. Only interactions with a confidence score > 0.9 were retained to ensure high reliability. The resulting network was visualized using cytoscape software (**Figure 2E**). The Molecular Complex Detection (MCODE) plugin was applied to identify functionally significant gene clusters. Three tightly connected gene modules were ultimately identified (**Figures 2F–H**). The key gene *SP1* (specificity protein 1) we studied is in the third cluster.

### 3.4 Heatmap and tissue-specific expression profiling of top 50 DE-FRGs

**Figure 3:**
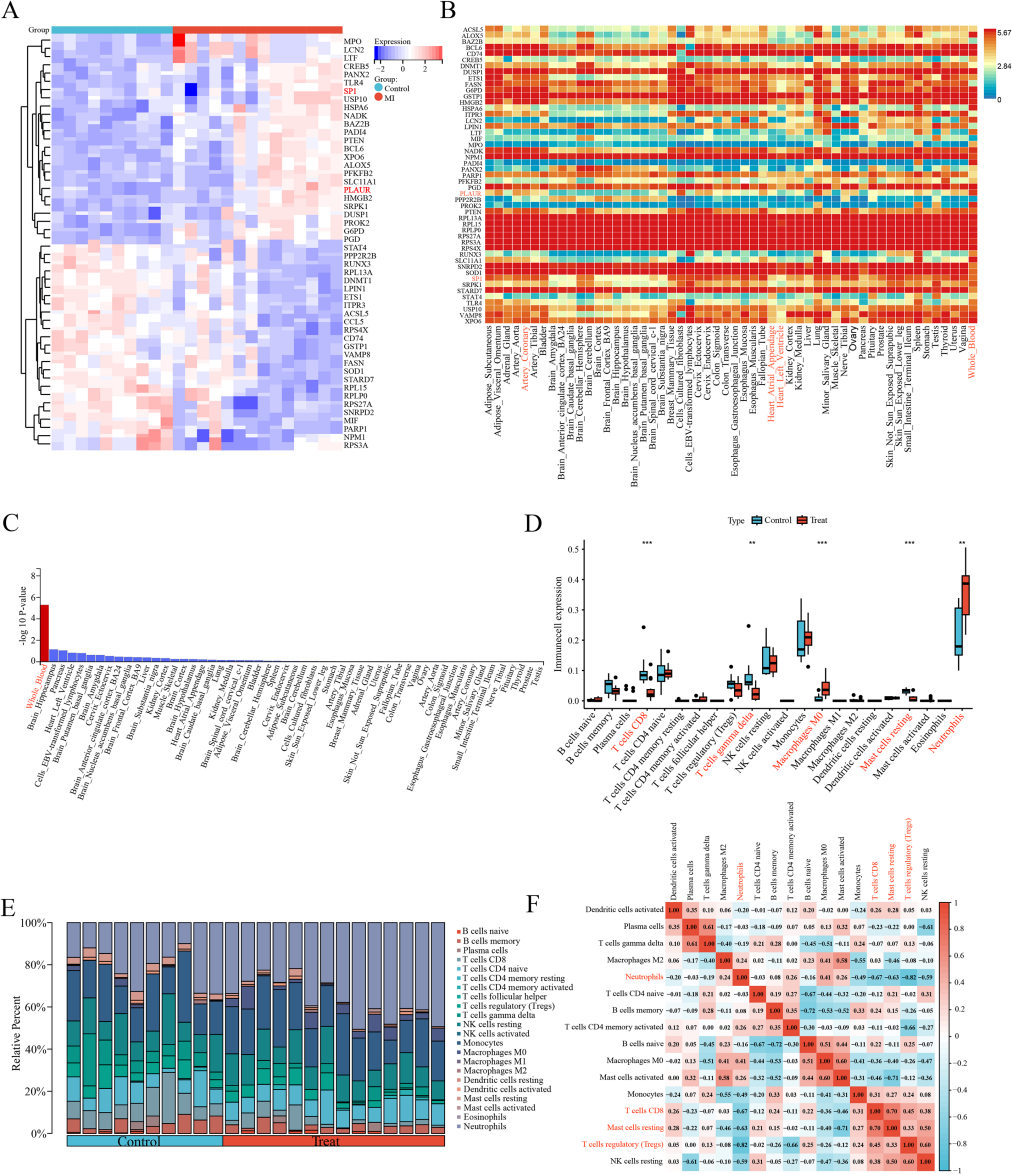
Gene-expression heatmap, tissue-specific expression, and results of immune infiltration analysis. **(A)** Expression heatmap for top 50 of 269 DE-FRGs. **(B)** Heatmap demonstrates tissue-specific expression patterns of top 50 DE-FRGs. Analysis was conducted using GENE2FUNC in FUMA, based on the GTEx v8 database comprising 54 tissue types. The x-axis represents different tissues, while the y-axis lists the genes analyzed. The color gradient corresponds to the expression levels, with blue indicating low expression, yellow indicating moderate expression, and red representing high expression. **(C)** Plot shows the differential expression of 269 genes in different tissues. **(D)** Box diagram displays different fractions of 22 immune cell sub-populations in MI and control samples. ** indicates statistical significance (*P* < 0.05) between MI and control groups. ** represents *P* < 0.01; *** represents *P* < 0.001. **(E)** The stack bar diagram displays the relative percent of 22 immune cell sub-populations in each sample. **(F)** Correlation heatmap of 22 immune cell sub-populations. The red represents positive correlation and the blue represents negative correlation.

The heatmap in **Figure 3A** illustrated the expression profiles of the top 50 DE-FRGs (ranked by fold-change) across control and AMI samples, clearly separated the two groups and provided an intuitive foundation for further gene-expression analyses. From the heatmap, it can be seen that the expression of the *SP1* and *PLAUR* (urokinase-type plasminogen activator receptor gene) is increased in AMI samples. Using FUMA-GENE2FUNC, we analyzed 50 DE-FRGs expression patterns across GTEx v8 tissues (**Figure 3B**). As shown in **Figure 3C**, these 50 genes exhibited differential expression across the four examined tissues: coronary artery, atrial appendage, left ventricle, and whole blood.

### 3.5 Immune infiltration analysis

The immune cell composition of the myocardium has been recognized as a critical determinant of infarct expansion and post-myocardial infarction remodeling. Through the box plot (**Figure 3D**), we gained valuable insights into the differential expression of various immune cells between the control and AMI samples. **Figure 3E** depicted the relative abundance of 22 immune cell subsets in each sample from GSE60993. A robust positive relationship was observed between CD8^+^ T cells and resting mast cells (r = 0.70), and a pronounced inverse association was found between neutrophils and regulatory T cells (r = -0.82) (**Figure 3F**), providing a comprehensive overview of the immune-cell interaction landscape following MI.

### 3.6 Genetic evidence identifies *SP1* as an AMI risk-associated gene

**Figure 4:**
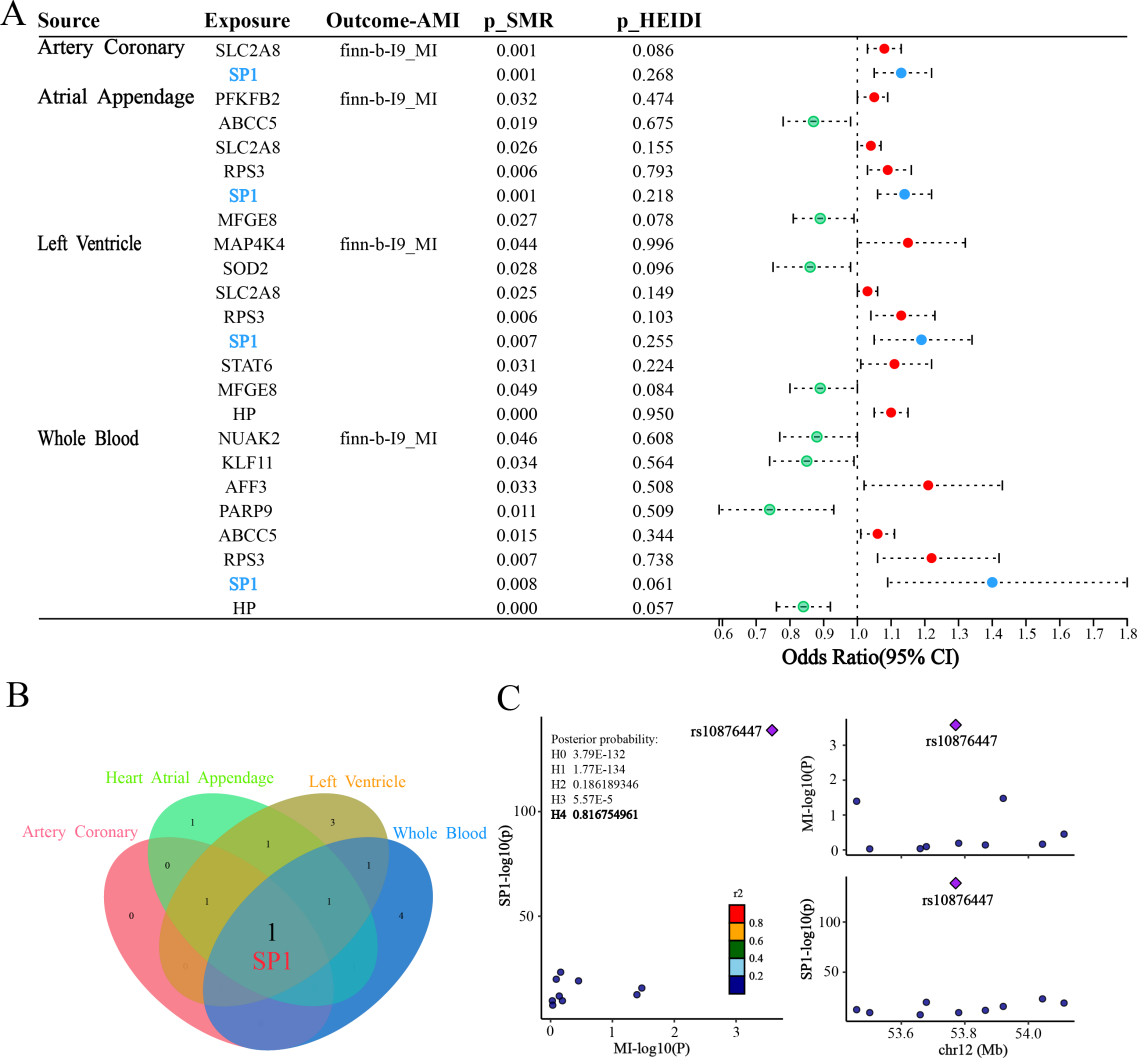
Results of SMR and colocalization analysis. **(A)** Forest plot shows the associations between 4 types of tissues with AMI. Red dots represent genes positively correlated with MI, green dots represent genes negatively correlated with MI, and blue dots represent significant genes common to four tissues. **(B)** Venn plot illustrates intersection of 4 types of SMR positive results. **(C)** Regional plot of colocalization evidence of *SP1* and MI.

SMR was employed to estimate the causal effects of the 269 DE-FRGs on AMI. For the finn-b-I9_MI outcome, the forest plot (**Figure 4A**) vividly displays the positive outcomes from the SMR analysis in four tissues [coronary artery (*p*SMR = 0.001, *p*HEIDI = 0.268, OR = 1.13, 95%CI = 1.05-1.22), atrial appendage (*p*SMR = 0.001, *p*HEIDI = 0.218, OR = 1.09, 95%CI = 1.03-1.16), left ventricle (*p*SMR = 0.007, *p*HEIDI = 0.255, OR = 1.19, 95%CI = 1.05-1.34) and whole blood (*p*SMR = 0.008, *p*HEIDI = 0.061, OR = 1.40, 95%CI = 1.09-1.80)]. Within the plot, red dots symbolize a positive correlation between the genes and AMI, potentially indicating genes that may promote the development of AMI. Green dots, on the other hand, represent a negative correlation, suggesting genes that could have a protective effect against AMI. Blue dots highlight genes whose SMR associations remained significant across four tissues, indicating robust, cross-tissue reproducible evidence for putative causality.

Subsequently, we employed a Venn diagram (**Figure 4B**) to visually represent the positive results obtained from the four SMR analyses. Through this graphical approach, we identified a common intersecting gene *SP1*. This finding suggests that *SP1* may exhibit a causal relationship with AMI across ventricular, atrial appendage, coronary artery and peripheral blood tissues.

Bayesian colocalization analysis provided strong evidence for shared genetic causality between the *SP1* locus and AMI. Specifically, the LocusZoom plot (**Figure 4C**) revealed a robust colocalization signal at rs10876447 (chr12), with a posterior probability of hypothesis 4 (PPH4 = 0.81), implicating *SP1* as a plausible mediator of genetic susceptibility to AMI.

**Figure 5:**
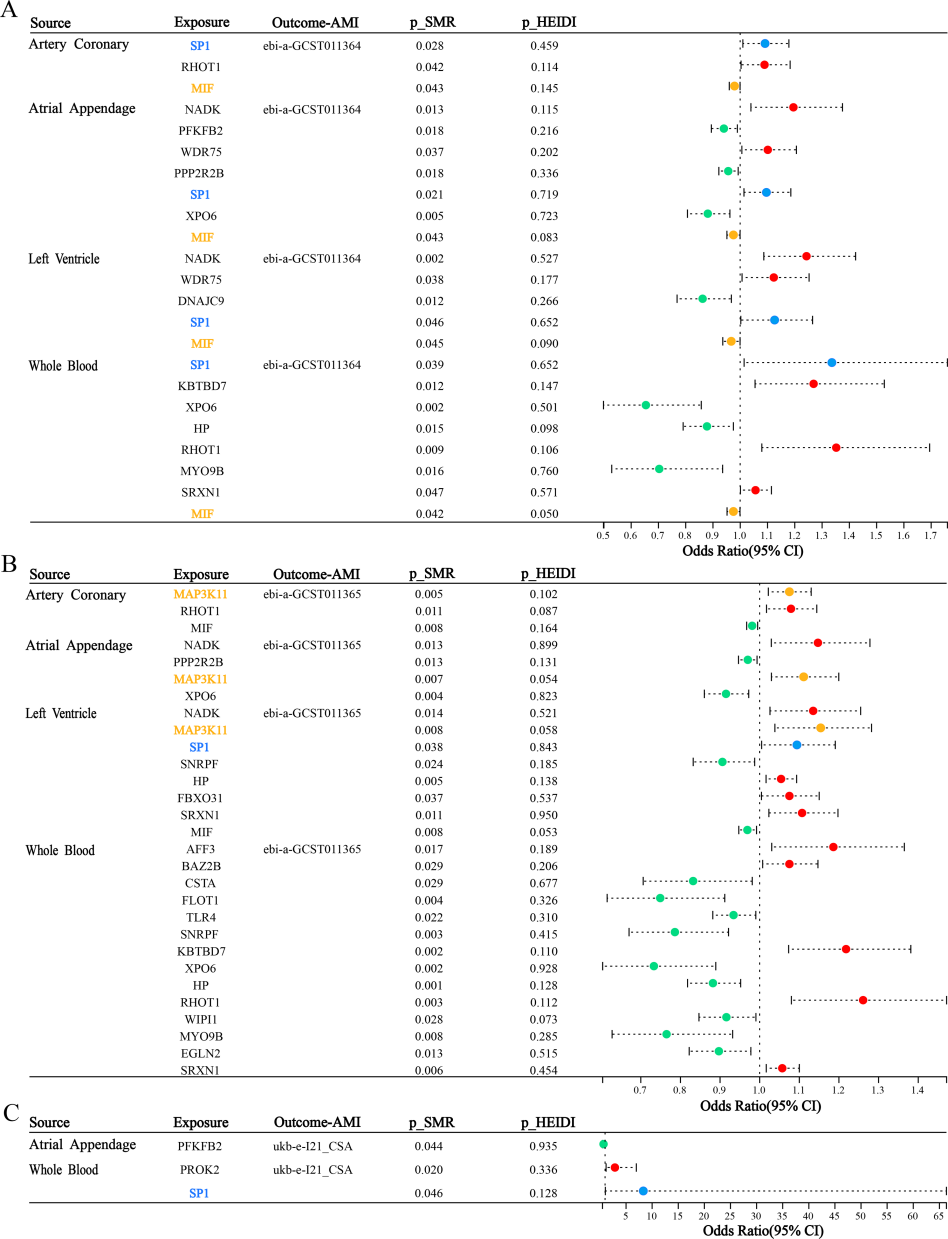
Forest Plot of SMR across three independent AMI outcome datasets. **(A)** Tissue-specific SMR associations between DE-FRGs and AMI (ebi-a-GCST011364). **(B)** Tissue-specific SMR associations between DE-FRGs and AMI (ebi-a-GCST011365). **(C)** Tissue-specific SMR associations between DE-FRGs and AMI (ukb-e-I21_CSA). Red dots represent genes positively correlated with MI, green dots represent genes negatively correlated with MI, and blue and yellow dots represent significant genes common to four tissues.

The ebi-a-GCST011364 dataset is used for replication validation, the forest plot (**Figure 5A**) vividly displays the significant outcomes from the SMR analysis in four tissues [coronary artery (*p*SMR = 0.028, *p*HEIDI = 0.459, OR = 1.091, 95%CI = 1.010-1.178), atrial appendage (*p*SMR = 0.021, *p*HEIDI = 0.719, OR = 1.09, 95%CI = 1.014-1.185), left ventricle (*p*SMR = 0.046, *p*HEIDI = 0.652, OR = 1.126, 95%CI = 1.002-1.265) and whole blood (*p*SMR = 0.039, *p*HEIDI = 0.652, OR = 1.336, 95%CI = 1.014-1.760)]. The replication analysis using the ebi-a-GCST011364 dataset confirms the association between *SP1* expression and AMI risk across all four tissues, with statistically significant SMR effects and non-significant HEIDI tests, indicating consistent evidence of a potential causal relationship. In this myocardial infarction dataset, we also identified another potential key gene—Macrophage Migration Inhibitory Factor (MIF). MIF exhibited OR values consistently below 1 across all four tissues, suggesting a potential causal relationship with AMI that is protective in nature. This finding provides a novel foundation for our future integrative research.

For the ebi-a-GCST011365 outcome, we can observe that *SP1* has a potential causal relationship with AMI in left ventricular tissue (*p*SMR = 0.038, *p*HEIDI = 0.843, OR = 1.094, 95%CI = 1.005-1.191) from the forest plot (**Figure 5B**), which is consistent with the results we expected from our study. Additionally, we identified another potential key factor—Mitogen-Activated Protein Kinase Kinase Kinase 11 (*MAP3K11*). The SMR results for *MAP3K11* were consistent across three tissues, suggesting that it may play an important role in the pathogenesis and progression of AMI.

For the ukb-e-I21_CSA outcome, forest plot visualization (**Figure 5C**) revealed a putative causal association between *SP1* and AMI in whole blood (*p*SMR = 0.046, *p*HEIDI = 0.128, OR = 8.308, 95%CI = 1.040-66.342), corroborating our expected findings.

### 3.7 Construction of miRNA-TF-mRNA network

**Figure 6:**
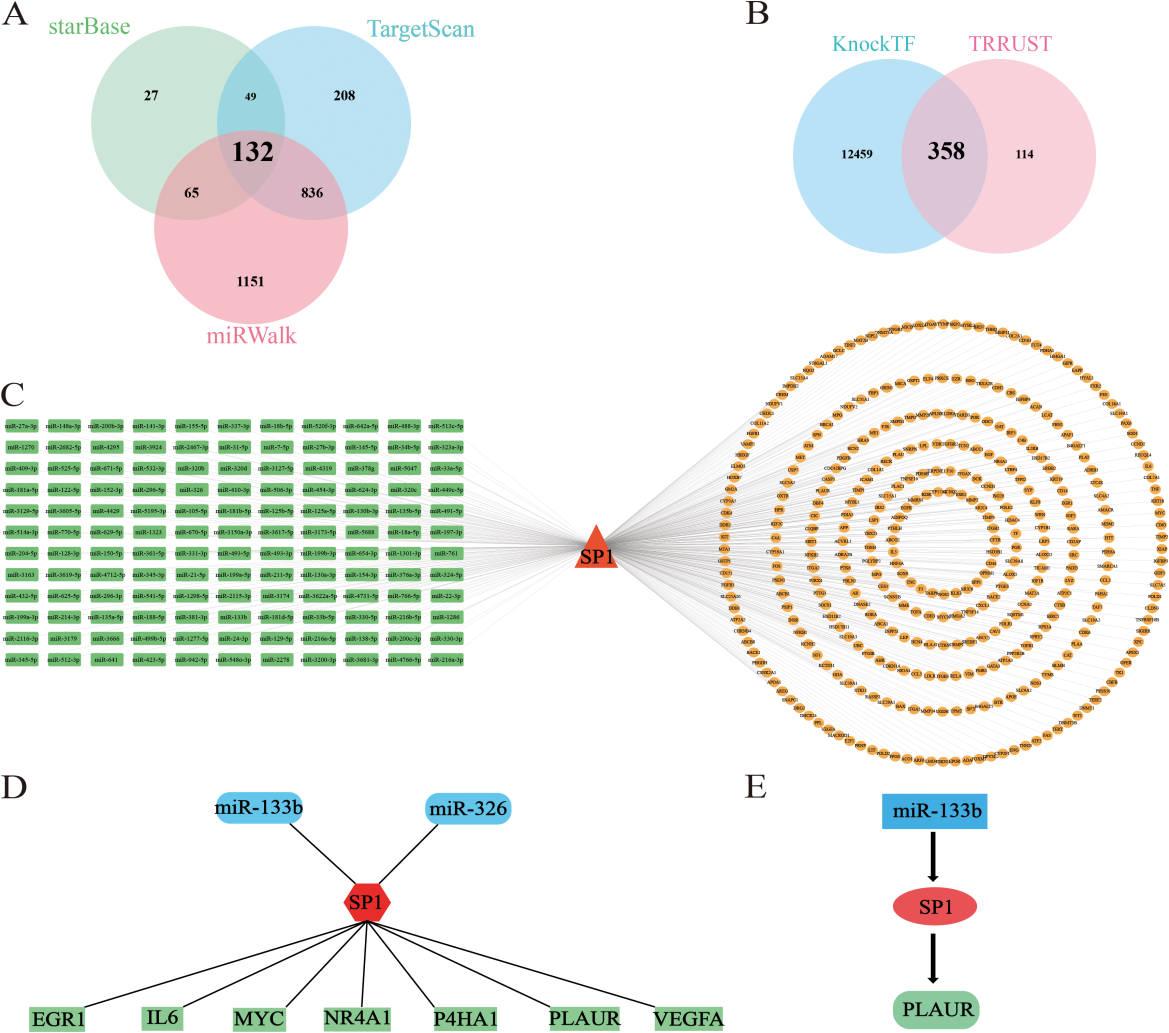
Construction of miRNA-TF-mRNA network. **(A)** Venn plot illustrates the results of predicting upstream miRNAs of *SP1* using starbase, mirwalk and targetscan together. **(B)** Venn plot shows the results of predicting down-stream mRNAs of *SP1* using TRRUST and KnockTF together. **(C–E)** miRNA-TF-mRNA network. C: red triangles, TFs; green squares, miRNAs; yellow ellipses, mRNAs. D–E: red, TFs; blue, miRNAs; green, mRNAs.

We focused on *SP1* as the core gene and used two or more online tools to jointly predict the upstream and downstream targets of it. The Venn diagram in **Figure 6A** illustrates the intersection of miRNAs predicted from three independent databases: miRWalk, TargetScan, and starBase. This integrative analysis identified 132 candidate miRNAs that were consistently predicted across three platforms. Furthermore, as demonstrated in **Figure 6B**, we conducted an integrative prediction of mRNAs regulated by *SP1*, leveraging data from two separate datasets: KnockTF and TargetScan. We identified 358 candidate mRNAs that were consistently projected as targets of *SP1* in both cohorts. Finally, we constructed the miRNA-TF-mRNA network as shown in the **Figure 6C**.

To refine our list of candidate genes, we initially conducted a differential expression analysis utilizing the GSE4648 dataset. Subsequently, we intersected the DE-mRNAs with ferroptosis-related genes as well as the 358 candidate genes. This intersection process ultimately yielded 7 candidate mRNAs (*EGR1*, *IL6*, *MYC*, *NR4A1*, *P4HA1*, *PLAUR* and *VEGFA*). In a parallel approach, employing the GSE76604 dataset for differential expression analysis, we similarly intersected the DE-miRNAs with ferroptosis-related miRNAs and the 132 candidate miRNAs. This strategy enabled us to pinpoint 2 miRNAs (miR-133b and miR-326) (**Figure 6D**). Given that miRNAs typically exert an inhibitory influence on their downstream target genes and *SP1* was up-regulated in AMI samples, we ultimately select miR-133b as the upstream regulatory factor. Finally, we successfully established the miR-133b/*SP1*/*PLAUR*/ferroptosis signaling pathway (**Figure 6E**).

**Figure 7:**
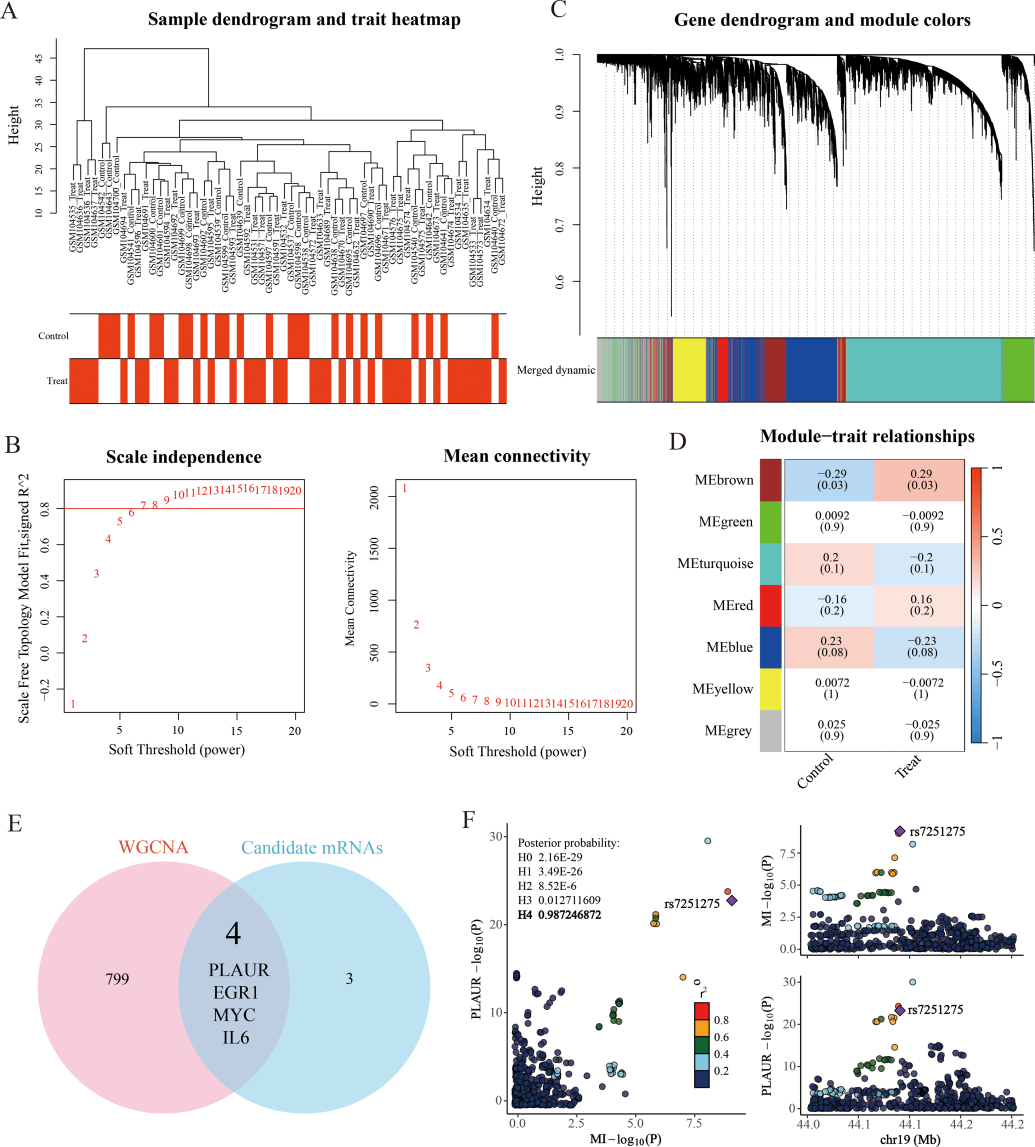
Weighted gene co-expression network (WGCNA) analysis. **(A)** Sample clustering and trait heatmap. **(B)** Scale-free soft threshold distribution. **(C)** Merging of module genes with traits and hierarchical clustering of genes into different modules (color-coded, with gray indicating unclassified genes). **(D)** Heatmap showing module correlation with clinical traits. **(E)** Venn diagram illustrates the intersection between 7 down-stream mRNAs of *SP1* and 803 genes positively correlated with MI in WGCNA analysis. **(F)** Regional plot of colocalization evidence of PLAUR and MI.

Following the previous steps, we conducted a weighted gene co-expression network analysis (WGCNA) using the GSE4648 dataset. Sample clustering revealed no outliers and supported reliable group separation (**Figure 7A**). A scale-free topology was obtained by choosing a soft-thresholding power of β = 10, which yielded a scale-free fit index R² > 0.8 while keeping the mean connectivity low (**Figure 7B**). After applying dynamic tree cut (MEDissThres = 0.25), closely related modules were merged, giving seven final modules (**Figure 7C**). A trait–module correlation heatmap showed that the brown module (MEbrown) had the strongest positive association with AMI (cor = 0.29, P < 0.05) (**Figure 7D**). This module was therefore designated as the AMI-related module and contained 803 genes for further analysis.

Subsequently, we intersected the 7 candidate mRNAs with the 803 key module genes to obtain 4 key genes (**Figure 7E**). Then, we performed a colocalization analysis of 4 candidate mRNAs with MI GWAS data from the Finnish database. Through this analysis, we identified that only *PLAUR* (plasminogen activator urokinase receptor) demonstrated strong colocalization evidence with acute myocardial infarction (PPH4 = 0.99). It was the same data as the outcome data in the SMR analysis, which added the reliability of our findings. The visual evidence for this finding was provided in the (**Figure 7F**).

Ultimately, through a series of rigorous and systematic analyses, including differential expression analysis, SMR, colocalization validation and regulatory relationship determination, we successfully established the miR-133b/*SP1*/*PLAUR*/ferroptosis signaling pathway. The construction of this pathway is based on robust evidence from various bioinformatics approaches, providing a comprehensive framework for understanding the molecular mechanisms of ferroptosis regulation in the context of acute myocardial infarction.

### 3.8 Binding sites prediction of the miR-133b/*SP1*/*PLAUR* axis

**Figure 8:**
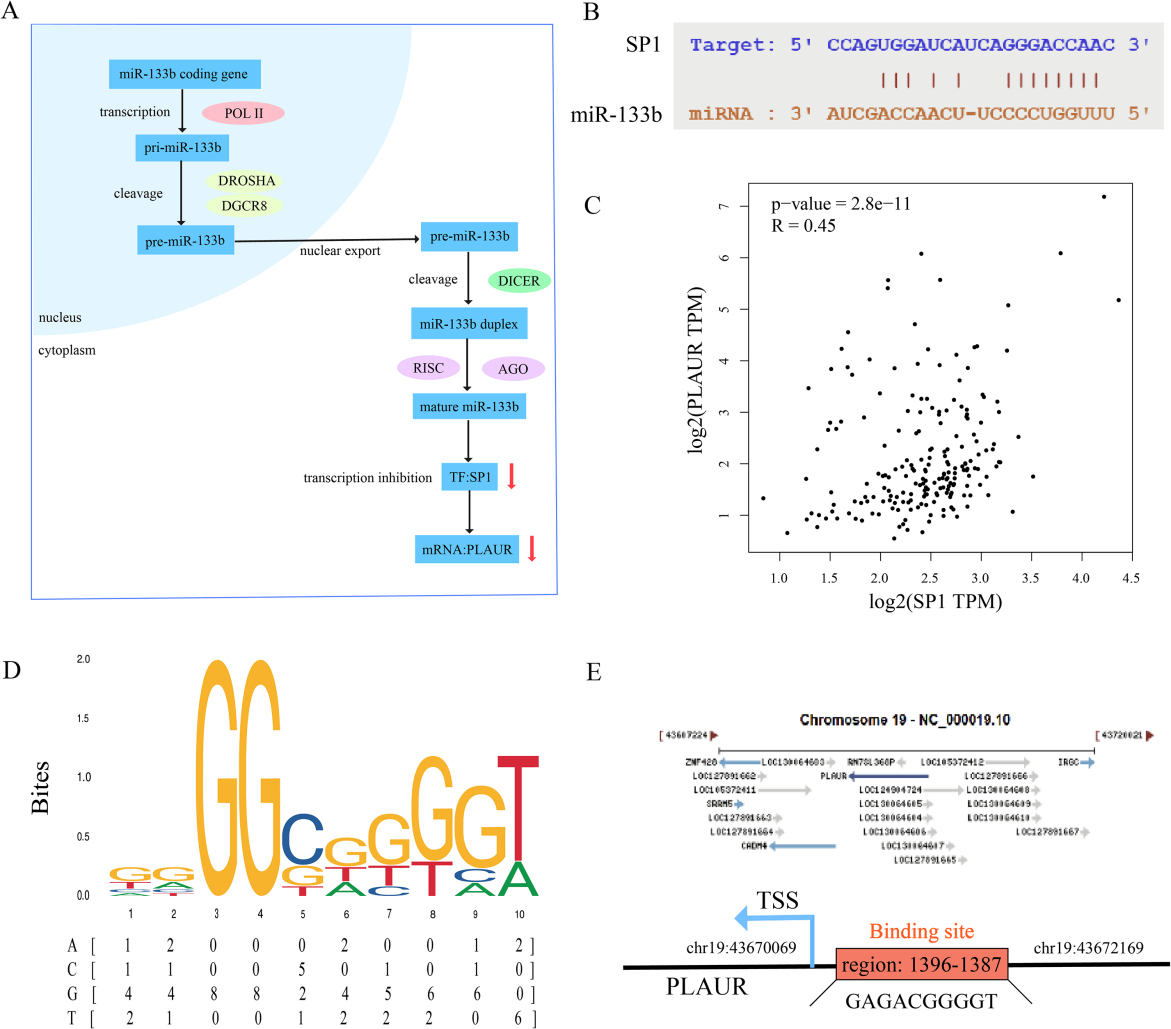
Binding site prediction of miR-133b/*SP1*/PLAUR axis. **(A)** Physiological mechanism of miR-133b/*SP1*/PLAUR axis. **(B)** Binding site of miR-133b and *SP1*. **(C)** Correlation analysis of *SP1* and PLAUR. **(D)** Binding motif of *SP1* predicted by JASPAR database. **(E)** Binding site of *SP1* and PLAUR.

The regulatory mechanism of the miR-133b/*SP1*/*PLAUR* axis within cells under physiological conditions is shown in **Figure 8A**. Under physiological conditions, miR-133b constrains the transcription factor *SP1*, thereby silencing *PLAUR* transcription. However, this regulatory axis is disrupted during AMI. Based on this, our study first proposes the following mechanistic hypothesis: in the ischemic and hypoxic microenvironment of the myocardium, miR-133b is initially downregulated, relieving its inhibition on the transcription factor *SP1*. The enhanced activity of *SP1* subsequently upregulates *PLAUR* expression. High expression of *PLAUR* mediates lipid peroxidation damage in cardiomyocytes by activating the ferroptosis pathway, ultimately exacerbating myocardial tissue necrosis and deterioration of cardiac function.

To validate the authenticity of the miR-133b/*SP1*/*PLAUR* axis, we first systematically reviewed the literature and confirmed that there are conserved binding sequences between miR-133b and the *SP1* 3’UTR, as well as between the *SP1* promoter and the *PLAUR* promoter region (42–46). Subsequently, we used online tools for interaction prediction and the results were fully consistent with the literature, providing a reliable sequence basis for subsequent functional experiments. First, using the starBase database, the complementary binding sites between miR-133b and the *SP1* were predicted, and the resulting binding sequence is shown in **Figure 8B**. Pearson correlation analysis was conducted using the GEPIA2 database (GTEx left ventricular tissue), and the results showed a significant positive correlation between *SP1* and *PLAUR* expression levels (r = 0.45, *P* = 2.8e-11, **Figure 8C**), suggesting that *SP1* may be involved in the transcriptional regulation of *PLAUR*. Subsequently, the *SP1* position-weight matrix (MA0079.1) was downloaded from JASPAR (**Figure 8D**), and motif scanning was performed within 2000 bp upstream and 100 bp downstream of the *PLAUR* transcription start site (TSS) with a relative profile score threshold ≥ 0.8. A total of 9 potential binding sites were identified, among which the site at chr19: 43670069–43672169 had the highest score (0.85), suggesting that *SP1* may directly bind to the *PLAUR* promoter region and up-regulate its expression (**Figure 8E**).

### 3.9 GeneMANIA/GSEA analysis of the *SP1/PLAUR* axis

**Figure 9:**
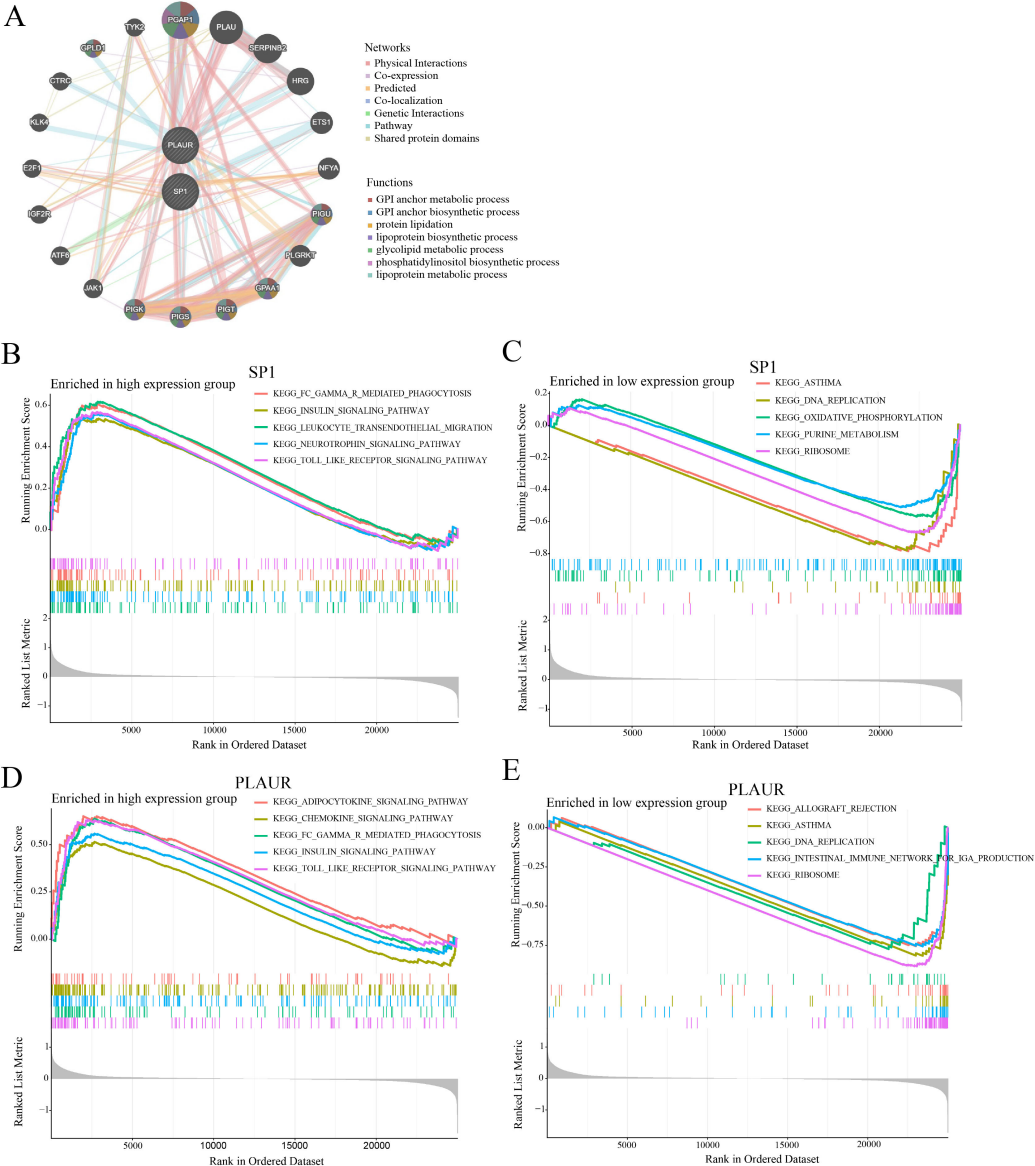
GeneMANIA and GSEA analysis results of *SP1* and PLAUR. **(A)** Result of GeneMANIA analysis. **(B-E)** Results of GSEA analysis.

GeneMANIA (**Figure 9A**) pinpointed 20 genes tightly linked to *SP1* and *PLAUR*, among which post-GPI attachment to proteins inositol deacylase 1 (*PGAP1*) exhibited prominent connectivity. Moreover, these nodes exhibited mutual interconnections, thereby giving rise to a highly intricate and complex network. Through GeneMANIA analysis, it was found that the studied genes are interconnected with a series of lipid - associated functions, including the metabolic and biosynthetic processes of GPI anchors, protein lipidation, lipoprotein biosynthesis and metabolism, along with glycolipid and phosphatidylinositol metabolism.

In this study, GSEA was employed to identify key signaling pathways potentially involving *SP1* and *PLAUR* in AMI. Enrichment plots revealed bidirectional pathway activity contingent on *SP1* abundance in AMI: high *SP1* expression (**Figure 9B**) was concomitant with the significant activation of five immune- and stress-related signaling pathways (FC-gamma-R-mediated phagocytosis, insulin signaling, leukocyte transendothelial migration, neurotrophin signaling and Toll-like receptor signaling), whereas *SP1* down-regulation (**Figure 9C**) preferentially engaged five metabolic or structural modules (asthma, DNA replication, oxidative phosphorylation, purine metabolism, and ribosome). GSEA disclosed a *PLAUR*-dependent switch in AMI-related pathway activity: elevated *PLAUR* (**Figure 9D**) selectively enriched pro-inflammatory and metabolic signaling circuits (adipocytokine, chemokine, FC-gamma-R-mediated phagocytosis, insulin and Toll-like receptor pathways), whereas *PLAUR* down-regulation (**Figure 9E**) shifted the transcriptional landscape toward immune-dysregulation and biosynthetic programs (allograft rejection, asthma, DNA replication, intestinal immune network for IgA production and ribosome).

### 3.10 Prediction of gene-drug and gene-disease

**Figure 10:**
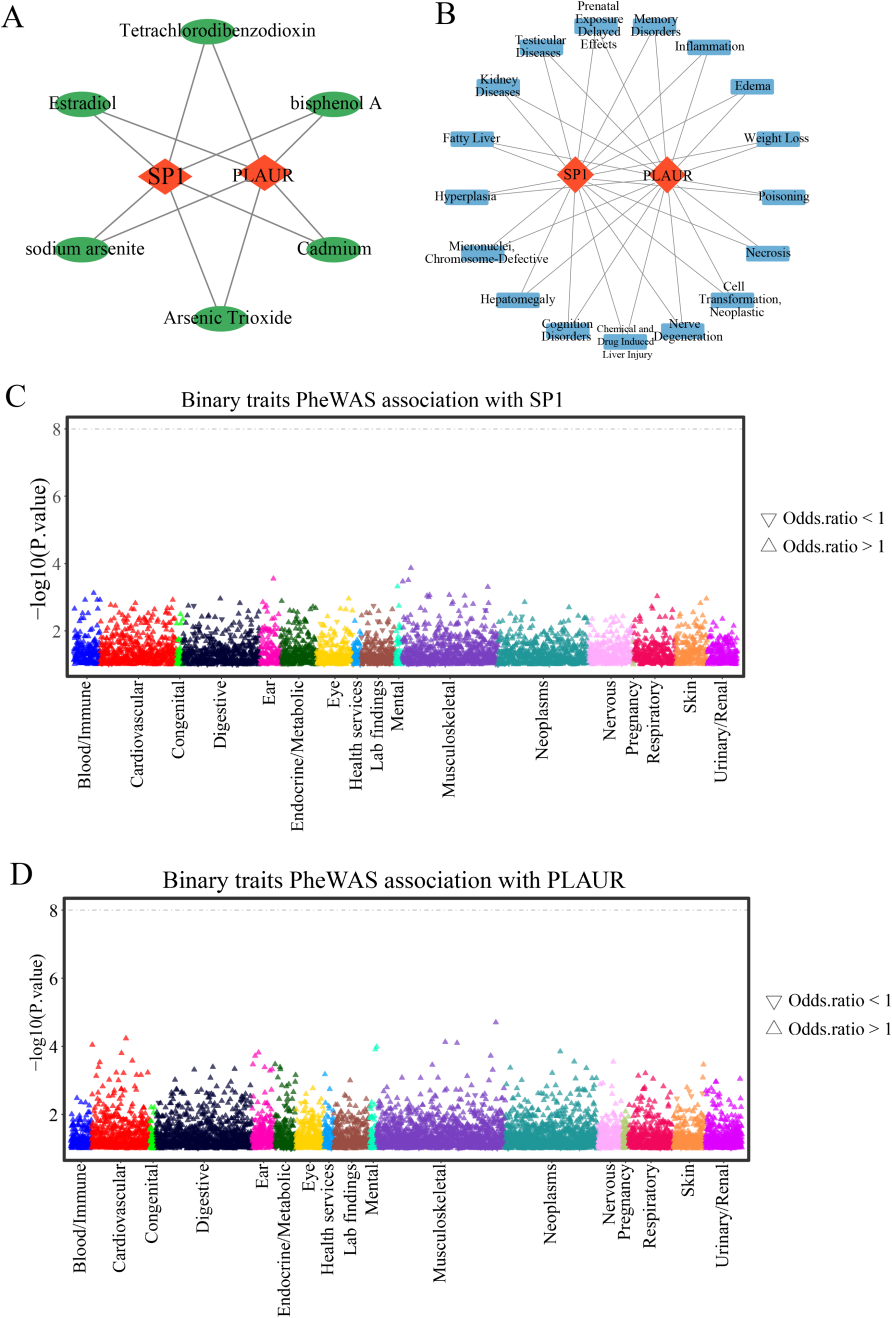
Gene-drug and gene-disease prediction and PheWAS. **(A)** Gene-drugs networks predicted by the CTD database. **(B)** Gene-diseases networks predicted by the CTD database. **(C)** Binary traits association with *SP1*. **(D)** Binary traits association with PLAUR.

In the CTD Database, candidate drugs and diseases associated with *SP1* and *PLAUR* were retrieved independently. For each gene, compounds and disease entries were ranked by their CTD Inference Score, and the top 20 drugs and diseases were retained. The two ranked drug lists and the two ranked diseases lists were then intersected, yielding six small-molecule compounds (**Figure 10A**) and seventeen diseases (**Figure 10B**) that are jointly targeted to both *SP1* and *PLAUR*, thus prioritizing dual-targeting candidates for further investigation. Through this strategy, we identified the potential dual-target intervention candidates, providing direction for subsequent AMI mechanism research and drug development.

### 3.11 PheWAS analysis

Using the PheWAS analysis, we scanned for phenotypes linked to *SP1* or *PLAUR*. No associations reached genome-wide significance (*P* < 5 × 10^-8^) (**Figures 10C, D**), indicating that therapeutic targeting of *SP1* or *PLAUR* is expected to carry minimal adverse drug reactions or unintended horizontal pleiotropy.

### 3.12 Evaluating the diagnostic value of miR-133b, *SP1* and *PLAUR* as biomarkers for AMI

**Figure 11:**
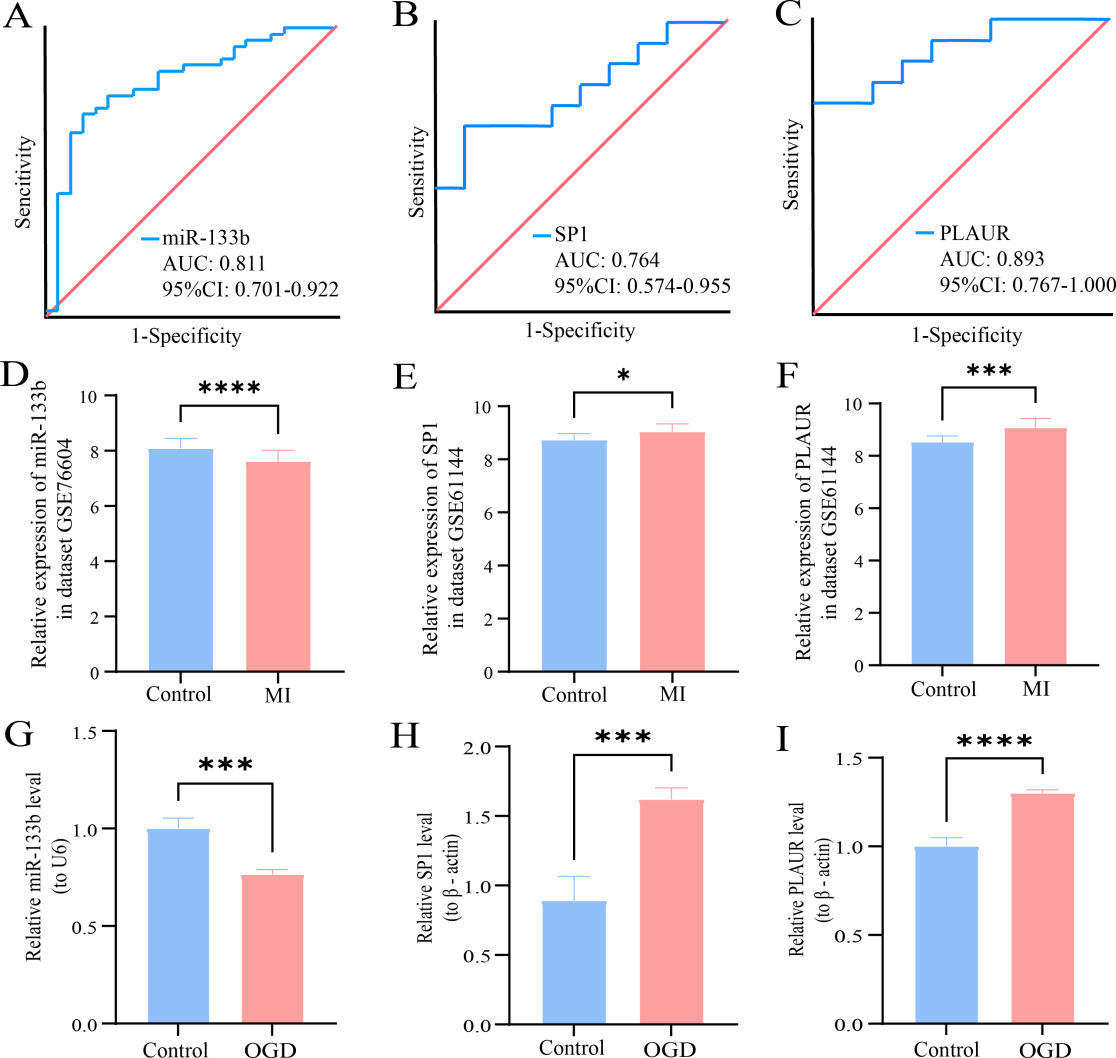
ROC curve and RT-qPCR of three biomarkers. **(A-C)** Receiver operating characteristic (ROC) curve for miR-133b, *SP1* and PLAUR. **(D-F)** The bar chart represents the expression of miR-133b, *SP1* and PLAUR in the dataset GSE76604 and GSE61144. **(G-I)** The qRT-PCR results of miR-133b, *SP1* and PLAUR in H9C2 cardiomyocytes. * indicates statistical significance (*P* < 0.05) between MI and control groups. ** represents *P* < 0.01; *** represents *P* < 0.001; ****V represents *P* < 0.0001. MI, myocardial infarction; OGD, oxygen-glucose deprivation model.

The receiver operating characteristic (ROC) curves were generated to evaluate the discriminatory power of the genes in distinguishing AMI cases from non - AMI individuals. The area under the curve (AUC) values were calculated to quantify the overall diagnostic accuracy. A higher AUC value indicates a better ability of the biomarker to correctly classify AMI patients and healthy controls. Through this analysis, the ROC curve (**Figure 11A**) showed that the AUC of miR-133b was 0.811 (95% CI = 0.701–0.922, *P* = 0.000). The ROC curve (**Figures 11B, C**) showed that the AUC of *SP1* was 0.764 (95% CI = 0.574–0.955, *P* = 0.030) and the AUC of *PLAUR* was 0.893 (95% CI = 0.767–1.000, *P* = 0.001). In summary, the ROC curve analyses of miR-133b, *SP1*, and *PLAUR* using data from GSE76604 and GSE61144 datasets, respectively, offer valuable insights into their diagnostic potential as biomarkers for AMI.

### 3.13 Experimental validation of miR-133b, *SP1* and *PLAUR* expression by RT-qPCR

**Figures 11D–F** displayed the expression levels of miR-133b, *SP1* and *PLAUR* in both the GSE76604 and GSE61144 datasets. In AMI group, miR-133b was significantly downregulated, whereas *SP1* and *PLAUR* were upregulated, consistent with the differential expression analysis and SMR findings. These results were further validated by pRT-PCR experiments in H9C2 cardiomyocytes subjected to hypoxia-induced injury, which showed the same expression trends (**Figures 11G–I**).

## 4 Discussion

**Figure 12:**
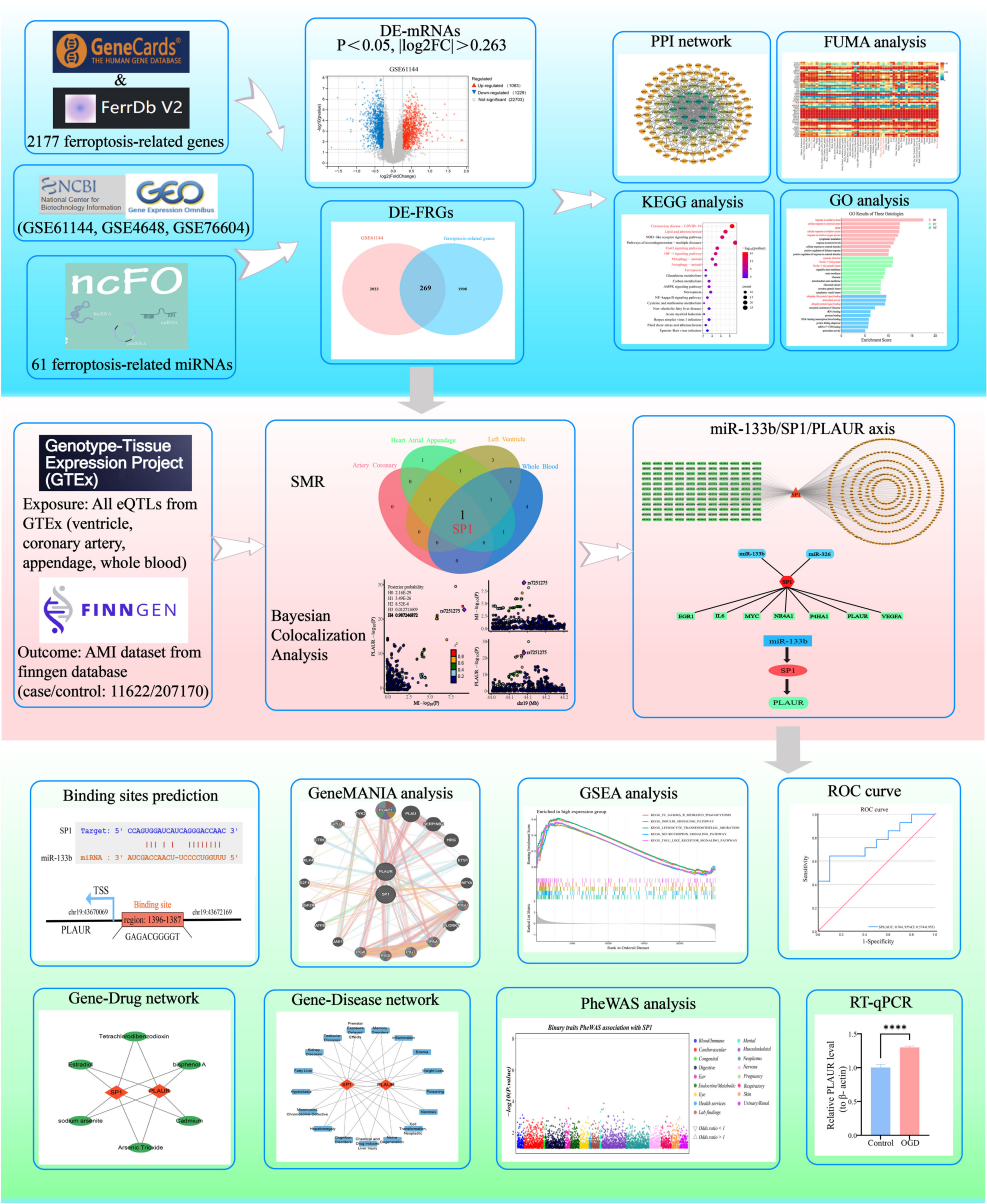
Integrated schematic of study design and key findings.

AMI remains a leading global health threat, exacting a heavy burden in both high and low-income nations. Rapid recognition, prevention, accurate diagnosis and prompt intervention are therefore critical to curb its worldwide mortality. Ferroptosis has emerged as a critical driver of AMI by accelerating the collapse of membrane integrity and precipitating cell lytic death, thereby amplifying infarct expansion and cardiac dysfunction (47). However, biomarkers capable of early warning and precise diagnosis of ferroptosis in AMI, as well as therapeutic targets with druggability potential, remain critically scarce. Here, we systematically integrated high-throughput transcriptomic public datasets (GSE61144, GSE4648 and GSE76604) to identify some robust DE-FRGs that reliably distinguish AMI patients from healthy controls. Using multidimensional bioinformatics analysis, combined with SMR, Bayesian colocalization and WGCNA analysis, we systematically analyzed the regulatory networks of these biomarkers and evaluated their causal contributions to AMI, ultimately identifying some key protein nodes to provide a scientific basis for subsequent precise targeted interventions. **Figure 12** shows the design flowchart of this study and an overview of the main research results.

This study identified 269 DE-FRGs from 2,292 DE-mRNAs and placed the ferroptosis within the context of lipid metabolic disorder and oxidative stress in AMI. KEGG enrichment simultaneously indicated four pathways: “lipid metabolism and atherosclerosis”, “FoxO”, “HIF-1” and “mitophagy” suggesting that ferroptosis is not an isolated event but rather an intersection point of lipid signaling, energy crisis, and immune activation in a hypoxia-reperfusion environment (48, 49). In the GO results, “antioxidant activity” and “ubiquitin-like ligase binding” were significantly co-enriched, indicating that beyond the classic *GPX4* axis, the ubiquitination system may constitute another gate regulating the susceptibility of cardiomyocytes to ferroptosis (50, 51).

Specificity protein 1 (*SP1*), identified as the pioneer transcription factor in mammals (52), exhibits widespread expression across nearly all tissues, with particularly high levels in the lungs, muscles and heart (53). *SP1* participates in multiple biological pathways and is closely linked to the development of breast, gastric and lung cancers (54). Emerging studies show that *SP1* also drives cardiac pathogenesis by governing cardiomyocyte death, fibrotic remodeling, inflammatory signaling and vascular calcification (55, 56). *SP1* can either activate or repress gene transcription, thereby modulating both protein-coding transcripts and non-coding RNA expression. *SP1*, a highly conserved zinc-finger factor, amplifies ferroptosis by docking on the *ACSL4* promoter, transcriptionally boosting the enzyme that drives lipid peroxidation and cell death (57, 58). Existing research indicates: bioinformatics analyses have suggested a potential causal relationship between *SP1* and AMI, with diagnostic value for AMI risk prediction (59). Mechanistically, *SP1* has been reported to mediate OGD/R-induced cardiomyocyte injury by enhancing *USP46* transcription (60), while targeting *SP1*-PARP1 signaling may protect against myocardial ischemia-reperfusion injury via autophagy downregulation (61). In this study, integrating tissue-specific SMR with Bayesian colocalization (PPH4 = 0.81), we established *SP1* as a high-confidence causal locus for AMI. The consistent SMR evidences across coronary artery, atrial appendage, ventricular myocardium and peripheral blood indicated that *SP1*-driven AMI risk is not confined to a single cardiac compartment but is systemically encoded. This trans-tissue evidence implied that *SP1* promote AMI susceptibility through a network mechanism. This study revealed for the first time a potential causal association between *SP1* and the risk of AMI at the genetic level through Mendelian randomization, and obtained stable and consistent positive evidence in four cardiovascular-related tissues.

This study centered on *SP1* and constructed a regulatory network encompassing “miRNA-TF-mRNA”: upstream, *SP1* expression was regulated by miR-133b and miR-327; downstream, *SP1* directly transcriptionally activated seven core target genes: *EGR1*, *IL6*, *MYC*, *NR4A1*, *P4HA1*, *PLAUR* and *VEGFA*. These genes mediate processes such as inflammation, glucose and lipid metabolism, collagen modification, and vascular permeability, ultimately driving the ferroptosis program in cardiomyocytes. Studies have shown that miR-133b expression is reduced after myocardial infarction and is associated with myocardial cell fibrosis, which is consistent with the results obtained in this study (62, 63). Downregulation of miR-327 can reduce the production of reactive oxygen species (ROS) and intrinsic apoptosis, and alleviate I/R injury (64). Recent studies have confirmed the existence of an independent *EGR1*/miR-15a-5p/*GPX4* signaling axis in MI (65). This study predicts that the promoter region of *EGR1* contains high-confidence *SP1* binding sites, suggesting that *SP1* may directly drive *EGR1* expression, and can also antagonize ferroptosis through the *EGR1*/miR-15a-5p/*GPX4* pathway, forming a ferroptosis switch with coexisting positive and negative feedback, which is worth our further exploration. *MYC* overexpression improves post-MI recovery in mice by promoting cardiomyocyte proliferation, suggesting that *SP1/MYC* axis may offer a new therapeutic angle for better prognosis (66). Studies have reported that *PLAUR* is significantly upregulated during ferroptosis (67), which is consistent with our results; however, the specific downstream pathways (such as the *GPX4*/*SLC7A11* axis, lipid ROS accumulation, or iron metabolism regulation) and molecular mechanisms through which it mediates ferroptosis remain to be systematically elucidated.

We were the first to identify the “miR-133b/*SP1*/*PLAUR*” axis as a druggable ferroptosis-amplifying signal pathway: ischemic stress markedly reduces miR-133b, thereby relieving its post-transcriptional repression of *SP1*; derepressed *SP1* directly occupies the *PLAUR* promoter and promote its transcription. Subsequent *PLAUR* up-regulation amplifies lipid peroxidation and activates the labile iron pool, culminating in cardiomyocyte ferroptosis. This positive-feedback loop provides a molecular basis for the rapid dissemination of ferroptosis during acute myocardial infarction and indicates that restoring miR-133b or interrupting the *SP1*/*PLAUR* transcriptional axis may constitute a promising strategy to limit ferroptosis-driven myocardial injury. As shown in **Table 4**, we contrast the miR-133b/SP1/PLAUR axis with the canonical System Xc^-^/GSH/GPX4 ferroptosis pathway.

**Table 4:** Comparative our study with the classical ferroptosis pathway.

Comparison Dimension	Our study (miR-133b/SP1/PLAUR axis)	Classical Ferroptosis Pathway (System Xc^-^/GSH/GPX4 axis)
Key Molecules	Transcription factor: SP1	Central regulator: GPX4
	Target gene: PLAUR (uPAR)	Cystine/glutamate antiporter: System Xc^-^ (SLC7A11/SLC3A2)
	Upstream miRNA: miR-133b	Enzymes: ACSL4, LPCAT3
Regulatory Mechanisms	miR-133b↓-SP1↑-PLAUR↑	System Xc^-^↓-GSH↓-GPX4↓
Relationship with Ferroptosis	SP1 promote ferroptosis	SLC7A11/SLC3A2 inhibit ferroptosis
	PLAUR promote ferroptosis	GPX4 inhibit ferroptosis
		ACSL4/LPCAT3 promote ferroptosis
Regulation Level	Upstream regulation (miRNA → transcription factor → membrane receptor)	Downstream effects (metabolic enzymes, antioxidant system)
Potential Crosstalk/Interactions	• SP1 may transcriptionally regulate ferroptosis-related genes (e.g., ALOX5) – (PMID: 9062372; 9062372)	
	• PLAUR-driven inflammation may alter redox/iron homeostasis, sensitizing cells to ferroptosis	
	• Both pathways converge on oxidative stress and tissue injury in AMI	

Of course, our study has several inherent limitations. First, the conclusions are primarily derived from bioinformatics analyses combined with *in vitro* cellular experiments, and thus lack longitudinal validation in animal models and human specimens. Second, how *PLAUR* precisely regulate ferroptosis, for example its downstream effectors, remains elusive. Third, beyond directly initiating *PLAUR* transcription, whether *SP1* concurrently modulates other core ferroptosis genes or immune-microenvironment components has not been systematically mapped. Therefore, future work should first, explore and verify the miR-133b/*SP1*/*PLAUR* axis in a rat AMI model to confirm its driver function *in vivo*; second, develop oligonucleotide mimics or small-molecule inhibitors targeting miR-133b, *SP1*, or *PLAUR* and evaluate their therapeutic potential for reducing infarct size and improving cardiac function. These efforts will comprehensively clarify the role of the miR-133b/*SP1*/*PLAUR* regulatory axis in AMI-related ferroptosis and accelerate the clinical translation of these therapeutic targets. Bioinformatics predictions and existing literature strongly support the existence of the *miR-133b/SP1/PLAUR* axis and its regulatory logic, but this study has not yet provided direct functional evidence for this pathway in cardiomyocyte ferroptosis. Due to the experimental conditions and time constraints at the current stage of research, the related mechanisms remain to be verified. We have identified this as a key focus and plan to systematically elucidate the role of this axis in ferroptosis through genetic interventions of miR-133b, *SP1*, or *PLAUR* in cardiomyocytes, combined with the detection of ferroptosis markers and a myocardial ischemia-reperfusion injury animal model. There is also a limitation of our study is the inability to account for potential sex-specific genetic effects, as publicly available GWAS summary statistics for acute myocardial infarction are not stratified by sex. Given the known influence of sex hormones and biological sex on cardiovascular risk, future sex-stratified GWAS are needed to elucidate whether genetic associations with AMI differ between males and females.

## 5 Conclusion

This study integrated bioinformatics, summary-data-based mendelian randomization, Bayesian colocalization, PheWAS and experimental validation to establish for the first time that *SP1* is a cross-tissue causal gene for AMI and miR-133b/*SP1*/*PLAUR* forms a new regulatory axis driving myocardial ferroptosis. This axis has interventional potential and diagnostic value. Targeting the restoration of miR-133b levels or jointly inhibiting the *SP1*/*PLAUR* signaling pathway may overcome the limitations of current antioxidant strategy against schemia and hypoxia injury, providing new biomarkers and dual-target drug candidates for precise prevention and treatment of AMI.

## Data Availability

The original contributions presented in the study are included in the article/**Supplementary Material**. Further inquiries can be directed to the corresponding author.
